# Polygenic contributions to performance on the Balloon Analogue Risk Task

**DOI:** 10.1038/s41380-023-02123-x

**Published:** 2023-08-15

**Authors:** E. L. Nurmi, C. P. Laughlin, H. de Wit, A. A. Palmer, J. MacKillop, T. D. Cannon, R. M. Bilder, E. Congdon, F. W. Sabb, L. C. Seaman, J. J. McElroy, M. R. Libowitz, J. Weafer, J. Gray, A. C. Dean, G. S. Hellemann, E. D. London

**Affiliations:** 1 Department of Psychiatry and Biobehavioral Sciences, University of California at Los Angeles, Los Angeles, CA 90024 USA; 2 Department of Psychiatry, University of Chicago, Chicago, IL 60637 USA; 3 Department of Psychiatry, University of California at San Diego, La Jolla, CA 92093 USA; 4 Institute for Genomic Medicine, University of California San Diego, La Jolla, CA 92093 USA; 5 Peter Boris Centre for Addictions Research, McMaster University and St. Joseph’s Healthcare Hamilton, Hamilton, ON L8S4L8 Canada; 6 Departments of Psychology and Psychiatry, Yale University, New Haven, CT 06520 USA; 7 Prevention Science Institute, University of Utah, Salt Lake City, UT 84112 USA; 8 Department of Neurobiology, University of Kentucky, Lexington, KY 40506 USA; 9 Department of Psychology, University of Kentucky, Lexington, KY 40506 USA; 10 Department of Psychology, University of Georgia, Athens, GA 30602 USA; 11 Department of Public Health, University of Alabama at Birmingham, Birmingham, AL 35294 USA; 12 Department of Molecular and Medical Pharmacology, University of California at Los Angeles, Los Angeles, CA 90024 USA

**Keywords:** Psychiatric disorders, Genetics

## Abstract

Risky decision-making is a common, heritable endophenotype seen across many psychiatric disorders. Its underlying genetic architecture is incompletely explored. We examined behavior in the Balloon Analogue Risk Task (BART), which tests risky decision-making, in two independent samples of European ancestry. One sample (*n* = 1138) comprised healthy participants and some psychiatric patients (53 schizophrenia, 42 bipolar disorder, 47 ADHD); the other (*n* = 911) excluded for recent treatment of various psychiatric disorders but not ADHD. Participants provided DNA and performed the BART, indexed by mean adjusted pumps. We constructed a polygenic risk score (PRS) for discovery in each dataset and tested it in the other as replication. Subsequently, a genome-wide MEGA-analysis, combining both samples, tested genetic correlation with risk-taking self-report in the UK Biobank sample and psychiatric phenotypes characterized by risk-taking (ADHD, Bipolar Disorder, Alcohol Use Disorder, prior cannabis use) in the Psychiatric Genomics Consortium. The PRS for BART performance in one dataset predicted task performance in the replication sample (*r* = 0.13, *p* = 0.000012, pFDR = 0.000052), as did the reciprocal analysis (*r* = 0.09, *p* = 0.0083, pFDR=0.04). Excluding participants with psychiatric diagnoses produced similar results. The MEGA-GWAS identified a single SNP (rs12023073; *p* = 3.24 × 10^−8^) near *IGSF21*, a protein involved in inhibitory brain synapses; replication samples are needed to validate this result. A PRS for self-reported cannabis use (*p* = 0.00047, pFDR = 0.0053), but not self-reported risk-taking or psychiatric disorder status, predicted behavior on the BART in our MEGA-GWAS sample. The findings reveal polygenic architecture of risky decision-making as measured by the BART and highlight its overlap with cannabis use.

## Introduction

The ability to make decisions in uncertain conditions that involve the balance between risk and reward is fundamental to success and survival, and high risk-taking behavior is common among individuals with certain neuropsychiatric disorders [1–6]. For these reasons, the biological bases of risk-taking behavior, including its neural underpinnings [7, 8] and genetic architecture [9, 10], have been a subject of recent interest. The propensity for or tolerance of risk can be evaluated using questionnaires, such as the DOSPERT Scale [11] and other surveys of individual preferences [12], while actual risk-taking can be measured using laboratory tests such as the Iowa Gambling Task [13], the Cambridge Gambling Task [14], and the Balloon Analogue Risk Task (BART) [3]. Importantly, different measures of risk-taking, both self-report and laboratory, are poorly correlated and may measure distinct underlying processes [15].

There is evidence that risk-taking is heritable. A twin study estimated the contributions of genes and environment to risk-taking propensity, using a scale that integrated seven domains of risk-taking, and found additive genetic but individually unique environmental influences [9]. Heritability estimates ranged from 29–55% for the different domains of risk-taking in a meta-analyses of twin studies [9]. In another twin study, which measured risk taking on the Iowa Gambling Task, a latent “decision-making” factor was identified, and genetic factors explained 35%, 20%, and 46% of the variance in in a sample that was tested longitudinally at three times during adolescent development [16].

Some progress in identifying specific genes contributing to risk-taking has been made in large genome-wide association studies (GWAS). Studies of self-reported risk-taking in very large samples from 23andMe and the UK Biobank, among others, have produced numerous associations [17–25]. Converging data from these analyses implicated Cell Adhesion Molecule 2 (*CADM2*), a neural cell-adhesion gene, in several risk-taking phenotypes [18, 20–22, 24] and extended to drug and alcohol use phenotypes [23, 25–28]. In a study of UK Biobank participants, self-evaluation as a “risk-taker” was associated with loci on chromosomes 3 (rs13084531, highlighting *CADM2*) and 6 (rs9379971) and shared significant genetic risk with schizophrenia, bipolar disorder, attention-deficit hyperactivity disorder, post-traumatic stress disorder, smoking and obesity [21]. The largest study of self-reported risk taking to date included over a million individuals and identified 99 risk loci [24], implicating genes involved in glutamatergic and GABAergic neurotransmission. Finally, an interaction between a variant in Phospholysine Phosphohistidine Inorganic Pyrophosphate Phosphatase *(LHPP)* and alcohol dependence moderated self-reported history of risky sexual behavior and was associated with brain circuitries previously implicated in the inhibition of risky behavior [29]. Overall, the results suggested that risk-taking is a complex trait that is highly polygenic—driven by many genetic variants of small effect. Additional complexity is introduced by the method of phenotype assessment, whether self-reported personality attributions or objective performance on a laboratory test.

In this study, we measured risk-taking behavior on the BART [3], a laboratory test that measures risk-taking under ambiguous conditions. We used a laboratory test to avoid bias that can influence self-report data, and selected the BART because its psychometric properties are well characterized [1, 3, 4, 8, 30, 31]. We administered the BART in two large independent studies that also provided genomic data. The BART consists of a series of choices in which both the potential for reward and the risk of loss increase. Human data exploring the genetic basis of performance on the BART is sparse, and adequately powered studies are lacking. A twin study found that genetic factors explained 47% of the variation in risk-taking on the BART by young adults [32]. A study of inbred strains of rats, performing an adapted version of the BART, demonstrated an approximately similar level of heritability, with about 55% of the variance attributable to heritability, and data consistent with a polygenic model [33]. Conversely, the BART showed no evidence of family-based heritability across extended pedigrees in a population isolate [34]. In a study of 66 healthy adults, a score combining functional variation across five genes affecting dopaminergic signaling (*DRD2*, *DRD3*, *DRD4*, *DAT1*, and *COMT*) was related to dorsolateral prefrontal cortical function during risky decision-making and to task earnings [35]. A candidate gene study of 223 youths reported that female but not male carriers of the *COMT*^158^Met allele had higher risk-taking propensity compared to Val homozygotes on a youth-adapted version of the BART [36]. Overall, these findings suggest that performance on the BART has a heritable component that may be related to dopaminergic function, but the results should be interpreted with caution given a propensity for bias in candidate gene studies.

Given variability in the existing literature, the present study sought to develop and test a polygenic model to estimate the variability in risky decision-making measured by the BART that is explained by common genetic variation, and to conduct a GWAS to identify specific risk variants. To increase generalizability and reproducibility, we applied the model in two large samples independently and tested for its association with psychiatric conditions known for risk-taking and impulsivity in large publicly available datasets.

## Methods

### Participants

Data for this study came from two projects. One was the Consortium for Neuropsychiatric Phenomics (CNP), a study performed at the Semel Institute of the University of California Los Angeles (UCLA) to examine underlying genetic and neural factors and their links to three neuropsychiatric illnesses: schizophrenia, bipolar disorder, and attention-deficit hyperactivity disorder (ADHD). Genetic, cognitive and behavioral data were similarly collected in the Genetics of Impulsivity (GOI) project, performed at the University of Georgia and the University of Chicago. Ancestry was self-reported and genetically confirmed in both studies. Combining the CNP and GOI samples increased power and improved generalizability of study results. Significant care was also taken in the analytic method (see Results section) to: a) ensure that results obtained in one sample generalized to the other sample; b) control for demographic differences between samples; and c) generalize findings to public datasets in the Psychiatric Genomics Consortium.

*CNP* Sample [37]. Healthy control participants, ages 21–50, were recruited by community advertisements in the Los Angeles area and were “White, not Hispanic or Latino;” or “Hispanic or Latino, of any racial group.” The primary language was either English or Spanish (*N* = 1138; 731 White, 407 Hispanic, of any race). Participants were excluded if they met the following criteria: neurological disease, history of head injury with loss of consciousness, use of psychoactive medications, and a positive drug screen on the day of testing. In addition, smaller samples of people with diagnoses of schizophrenia, bipolar disorder, and ADHD (following Diagnostic and Statistical Manual of Mental Disorders, Fourth Edition—Text Revision (DSM-IV-TR) [38]) were recruited using a patient-oriented strategy involving outreach to local clinics and online portals. Use of psychotropic medications was allowed in the patient groups. In total, 996 healthy individuals, 53 participants with schizophrenia, 42 with bipolar disorder, and 47 with ADHD were evaluated. Both healthy and affected individuals participating in the CNP study were included because, according to the model, we would expect a similar genetic basis in healthy and affected individuals, with more extreme phenotypes expressed in those with psychiatric disorders. Diagnoses for all individuals followed the DSM-IV-TR, and were based on the Structured Clinical Interview for DSM-IV (SCID-I) [39] supplemented by the Adult ADHD Interview (a structured interview form derived from the Kiddie Schedule for Affective Disorders and Schizophrenia, Present and Lifetime Version (KSADS-PL) [40]. Participants who were included underwent a neuropsychological battery and submitted blood samples for genotyping. All subjects gave written informed consent in line with the procedure approved by the Institutional Review Board at UCLA. Data from the CNP study have been reported in prior publications [41–57].

*GOI Sample* [15, 58]. A total of 934 Caucasian-ancestry participants 18–30 years of age were tested at two sites (40% at Athens, GA and 60% Chicago, IL). Inclusion criteria were English fluency, age 18–30 years, and self-reported Caucasian race and non-Hispanic ethnicity to minimize population stratification [59]. Exclusion criteria were scores >12 on the Alcohol Use Disorders Identification Test (AUDIT) [60] or the Drug Use Disorders Identification Test (DUDIT) [61]. All participants were screened for recent alcohol or drug use via breathalyzer or urine drug test before testing. Another exclusion criterion was treatment over the last 12 months or self-reported current need for treatment for: depression, bipolar disorder, general anxiety, social anxiety, post-traumatic stress disorder, obsessive compulsive disorder, panic attacks/disorder, phobia, schizophrenia spectrum disorders, anorexia, bulimia, or binge eating. ADHD was not excluded in this sample although it was exclusionary in the CNP sample. DNA was collected via a saliva sample for DNA collection in an Oragene DNA kit (DNA Genotek Inc., Kanata, ON, Canada).

#### Balloon Analogue Risk Task

The BART is a computerized behavioral measure of risky decision-making [4]. Virtual balloons are presented on a computer screen, one balloon per trial, and the participant can “pump” the balloons up by pressing a response key, virtually inflating the balloons. Each pump produces a set increase in an amount of money (e.g., 5 cents per pump) or points earned on that trial. However, after a certain number of pumps, determined probabilistically, the balloon explodes, and the trial yields no money or points. The participant must decide when to “cash out” of a given trial, by pressing a response key, to retain earnings in a cumulative bank. The objective is for the participant to earn as much money, or as many points, as possible across the trials in the task. Versions of the BART vary with respect to the number of trials/balloons used, as well as the probability of explosions (e.g., some tasks have used balloons with a single probability of explosion [4], while others have used different-colored balloons with different probabilities of explosion [3]). The primary dependent variable of the task is the mean or total number of pumps on trials in which the balloon did not explode; these have been termed ‘adjusted pumps’. The measure ‘adjusted pumps’ is preferred to the absolute number of pumps because explosions artificially restrict the range of pumping [30].

The CNP version of the BART task, programmed in E-Prime 2.0, consisted of 40 total trials, with balloons that were colored red or blue (20 of each color). Red balloons were “high risk”, with the probability of explosion on each red balloon randomly selected from a range of 1 to 32 pumps; blue balloons were “low risk”, in which the probability of explosion was randomly selected from a range of 1 to 128 pumps. The order of balloon color across trials was random. Participants received 5 points for each adjusted pump. The GOI version of the BART consisted of thirty balloons, associated with a probability of explosion selected from a range of 1 to 64 pumps. Participants in both studies did not receive payment for their performance.

#### Genetic analyses

Genotyping was performed using the Omni Illumina 500,000 SNP chip. For all genotype data, markers were excluded for quality control if they had less than a 95% genotyping rate, a minor allele frequency less than 1%, deviated significantly from Hardy Weinberg equilibrium (*p* < 10^−6^), or were identified as having non-random genotyping failure (*p* < 10^−10^). Individuals were excluded for missing genotypic data (<2% genotypes), missing phenotypic data, or deviation from expected autosomal heterozygosity (F_het_ < 0.2). To reduce spurious effects arising from poorly powered rare variants in these modestly sized samples, only SNPs with MAF greater than 0.20 were included in the analyses, thus emphasizing the inclusion of more reliable associations. Results were similar but slightly weaker when the traditional 0.01 cut-off was used. GWAS was performed on each of the CNP and GOI datasets as follows. Principal component analysis (PCA) was performed within study as well as joint with the 1000 Genomes (1KG) ancestry informative markers for use in QC and modeling efforts. Partial correlations (in R) were used in the polygenic scoring analysis to control for the population differences in the phenotype when comparing to the scored PCA-controlled GWAS. Plink [62] was used to perform two linear regressions with Mean Adjusted Pumps as the dependent variable of interest, supplying sex, age and the first five PCA dimensions as covariates (Mean Adjusted Pumps ~ sex + age + 5 ancestry principal components). Each set of summary statistics was clumped and, along with the paired genotypes from its complement study, used to create polygenic scores for each individual in the target sample [63]. As performed by the PRSice method, we tested multiple thresholds (in this case 500 possible thresholds between 0.001–0.5) by running a linear regression of the score at each threshold (MeanAdjPumpsZ~SCORE@THRESHOLD + sex + age + 5 ancestry principal components) to determine the optimal threshold (the smallest *p*-value). The *p*-value obtained at the optimal threshold is corrected for multiple testing (500 potential thresholds) using the false discovery rate (FDR). Scores were then compared using a partial correlation analysis that controlled for the same covariates in the target dataset as in the source’s GWAS. Imputation to 1KG Phase 3 was also performed on each dataset, and the same methodology was applied.

A MEGA-analysis GWAS was performed on the merged imputed genotypes of the CNP and GOI datasets. To account for the sample population differences, the MEGA-analysis included the population covariates of the respective sources while also covarying by the source factor itself. After standard QC measures (see methods above), PLINK was used to perform a linear regression per the following model (Mean Adjusted Pumps ~ gt + sex + age + study sample + 5 ancestry principal components). A quantile-quantile (Q-Q) plot of observed vs. expected *p*-values and Manhattan plot of the linear regression results were performed in R. Estimation of genetic variance of all SNPs was performed using the GREML method [64] as implemented in GCTA (v1.92.4) [65]. Risk scores were then derived and the best MEGA-PRS was then tested for overlap with the single question self-report of risk-taking (“Would you describe yourself as someone who takes risks?”) in European UK Biobank participants (*N* = 436,236) [19] and disease status in European samples from the 2017 public ADHD, Bipolar Disorder, Alcohol Use Disorder, and “ever/never” prior cannabis use datasets using PRS methods above. Public datasets representing Attention-Deficit Disorder (PGC & iPSYCH, *N* = 19,099 cases, 34,194 controls) [66] and Bipolar Disorder (PGC, *N* = 20,352 cases, 31,358 controls, effective sample size 46,582) [67], a non-psychiatric control phenotype (PGC Inflammatory Bowel Disease, which is a combination of Ulcerative Colitis and Crohn’s disease PGC datasets), as well as Alcohol Use Disorder (UK Biobank AUDIT, *N* = 121,604) [23] and prior cannabis use (UK Biobank and ICC, *N* = 53,179 cases, 131,586 controls, effective sample size 151,493) [28] were downloaded and summary statistics were extracted in order to construct PRS models. For each disorder, a PRS was constructed and tested for prediction of BART performance in our MEGA-analysis combined sample. Similarly, UK Biobank analyses were conducted using the summary statistics as reported by Clifton and colleagues [19] to evaluate a shared genetic propensity for risk-taking between self-report and BART performance in CNP, GOI and our MEGA samples according to the PRS methods above.

## Results

**Fig. 1:**
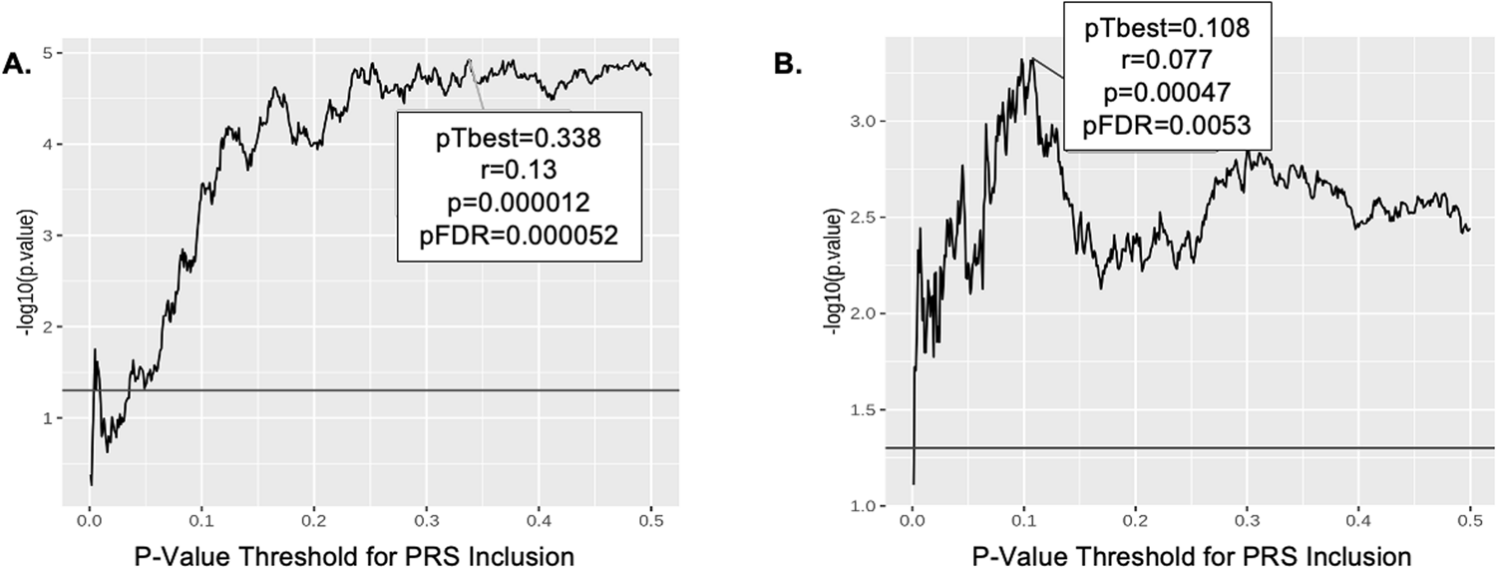
Polygenic Risk for Risky Decision Making as Measured by BART Performance is Replicated Across Independent Samples. **A** PRS analysis using GOI as training and CNP as replication sample. **B** PRS analysis using CNP as training and GOI as replication sample. The optimal threshold (0.338 in **A** and 0.108 in **B**), determined by strongest PRS correlation with BART phenotype, is highlighted by the box along with correlation (*r*), *p*-value, and FDR corrected *p*-value. Horizontal line indicates nominal significance.

A PRS for risky decision-making, constructed based on BART performance in the GOI sample (i.e., the discovery sample), predicted BART performance in the CNP sample (replication sample) (*r* = 0.13, *p* = 1.2 × 10^−5^), and the correlation remained significant (pFDR = 5.2 × 10^−5^) when corrected for multiple comparisons made in empirically determining the optimal *p*-value threshold (0.34) for SNP inclusion in the model (Fig. 1A). When the PRS was derived from BART performance in the CNP dataset and applied to the GOI sample, a similar correlation was observed (*r* = 0.09, *p* = 0.0083, pFDR = 0.04) at an optimal threshold of 0.361 (Fig. 1B). Exclusion of subjects with psychiatric diagnoses and Hispanic origin from the CNP data produced similar but less significant results, suggesting a power limitation.

**Fig. 2:**
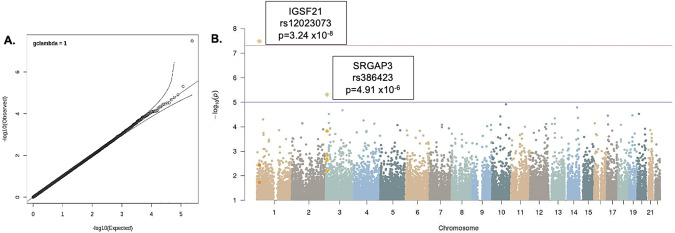
A GWAS Performed in the Combined GOI and CNP Samples Identifies One Locus Associated with BART Performance at the Genome-wide Significance Level. **A** Q-Q plot of expected vs. observed *p*-values for the MEGA-GWAS. **B** Manhattan plot of genome-wide association with risky decision-making as measured by BART performance. Upper line demarcates genome-wide significance. Lower line indicates Bonferroni correction significance threshold.

**Fig. 3:**
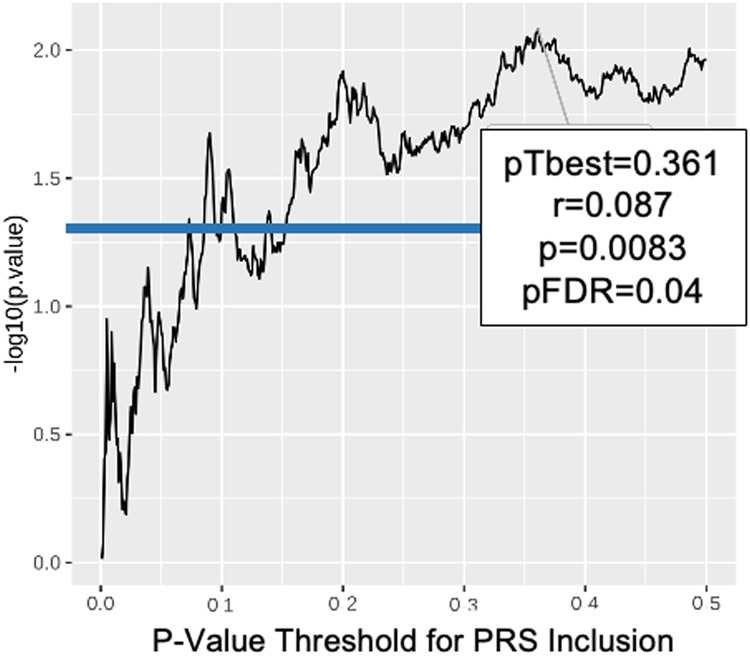
PRS Analysis of Cannabis Dependence Versus BART Performance in the Combined GOI and CNP Samples Reveals Shared Genetic Risk. The optimal threshold (0.11), determined by strongest PRS correlation with BART phenotype, is highlighted by the box along with correlation (*r*), *p*-value, and FDR corrected *p*-value. Horizontal line indicates nominal significance.

The Q-Q plot and genomic inflation factor *lambda* for the MEGA-analysis demonstrated that the principal components employed corrected for any effects of ancestry (Fig. 2A). MEGA-analysis linear regression (Fig. 3B) identified one variant that achieved genome-wide significance, SNP (rs12023073) in the first intron of immunoglobulin superfamily member 21 *(IGSF21)* on chromosome 1 (*p* = 3.24 × 10^−8^). The C-allele was associated with greater risk-taking compared to the minor T-allele (MAF = 0.34). A weaker signal occurred at rs386423 in a proximal intron of Slit-Robo GTPase activating protein 3 *(SRGAP3)* on chromosome 3 (MAF 0.41, *p* = 4.91 × 10^−6^). SNP heritability (h^2^ SNP) was significant at 0.27 (SE = 0.08, 7.1 × 10^−14^); however, this estimate should be interpreted with caution given the limitations of our small sample size.

When PRS data derived from the selected Psychiatric Genomics Consortium public datasets were applied to our MEGA-analysis, we observed a significant overlap in genetic factors underlying BART performance and prior cannabis use (Fig. 3, *p* = 0.00047, pFDR = 0.0053) but not ADHD, Bipolar Disorder, Alcohol Use Disorder or non-psychiatric control.

## Discussion

In line with the few previous rodent and human studies, our findings support a heritable, polygenic component to risky decision-making, with common variation (h^2^ SNP) explaining 27% of the variance in risk-taking on the BART. h^2^ SNP prediction in this modest sample (*n* = 2044) should be interpreted with caution [68]; however, our finding is in line with the 47% estimate from twin data that also includes rare variant contributions.

The MEGA-analysis identified two signals, one meeting a genome-wide significance threshold and the other approaching significance. Neither of these loci were reported by the large self-report biobank studies; however, consistent with our findings, gene pathway analysis in the largest study revealed an enrichment of brain expressed genes involved in maintaining the excitatory-inhibitory balance [24]. Our key associated SNPs map to genes that represent strong biological candidates for risk-taking and relevant phenotypic associations, as discussed below. The strongest signal from the MEGA-analysis occurred at SNP rs12023073, a variant in the first intron of *IGSF21*, annotated as impacting an enhancer of brain expression based on histone marks [69]. The immunoglobulin superfamily protein IGSF21, which is robustly expressed in brain tissue, is believed to play an integral role in thalamic and inhibitory synaptic development [70, 71]. Through an unbiased expression screen and proteomic analysis in mice, Tanabe and colleagues found that postsynaptic Igsf21 interacts with presynaptic Neurexin2α [70]. They further showed that *Igsf21* knockout mice have a number of phenotypic abnormalities, including impaired inhibitory presynaptic organization, diminished GABA-mediated synaptic transmission in hippocampal CA1 neurons, and deficits in sensory gating [70]. Interestingly, ethanol consumption increases the expression of *Igsf21* in rhesus macacques [72].

The rs386423 SNP in *SRGAP3*, demonstrating suggestive association, is annotated as an enhancer in multiple tissues based on histone marks, and is in perfect linkage disequilibrium with SNPs impacting brain expression [69]. Rodent studies reveal prominent hippocampal and cortical expression and gene knockout results in neurodevelopmental cognitive and behavioral phenotypes [73, 74]. In humans, SRGAP3 is also known as mental disorder-associated GAP protein (MEGAP) given its hypothesized role in chromosomal intellectual disability in the context of hemizygous loss of function [75]. Two de novo missense variants in *SRGAP3* were proposed to be related to Autism Spectrum Disorder in the Simons Simplex Collection [76].

Given the shift in conceptualizing mental illness from a categorical disease model to extremes of intersecting dimensional traits seen in the population [77], we tested whether risky decision-making represented one domain that would genetically overlap with psychiatric disorders characterized by prominent impulsivity and risk-taking. We hypothesized that ADHD, Bipolar Disorder, and substance use disorders (specifically Alcohol Use Disorder and cannabis use) would share polygenic underpinnings with risky decision-making, but only prior cannabis use was correlated with BART performance in our combined sample, notably withstanding correction for testing in 5 phenotypes. While a shared genetic basis between these phenotypes is also bolstered by the emergence of the *CADM2* locus as the strongest signal in multiple prior GWAS studies of both risk-taking [21, 24, 25] and Cannabis Use Disorder [26, 27], this locus did not contribute to risky decision making in our sample.

While our PRS findings suggest a common genetic contribution to both risky decision-making and cannabis use, we cannot isolate the component of behavior on the BART that is responsible for the genetic overlap. BART performance is a genetically complex phenotype. Because outcome probabilities are not known when the participant starts the task, multiple cognitive processes, which include the propensity for risk-taking as well as learning, are involved. In a study using a version of the BART similar to the one implemented in the CNP sample, adolescents who reported daily cigarette smoking failed to increase their responding to balloons across trials, whereas nonsmokers adapted their performance over time and thus earned more money [31]. This same study found that the adjusted pumps measure was modestly but positively associated with years of education and nonverbal IQ. At moderate levels, pumping on the task is adaptive and results in increased gains, despite the presence of some explosion trials. In contrast, excessive pumping is maladaptive. Relevant to the present finding with cannabis use is the observation that young adults who regularly use cannabis showed significant differences in self-reports on social, health/safety, and ethical risk-taking scales, but not in the propensity to take recreational or financial risks or in performance on a laboratory monetary risk-taking task, as compared non-using control participants [78]. Therefore, the component of BART performance that is linked to the observed genetic overlap with initiation of cannabis use is yet to be determined.

The complexity of the BART performance phenotype may also underlie the lack of genetic correlation with self-reported risk-taking in the large UK Biobank sample. The BART is an objective, quantitative measure but its complexity poses a barrier to achieving adequate sample sizes needed to detect smaller genetic effects. In contrast, the UK Biobank approach uses a very blunt tool but benefits from superior power. Thus, these approaches may both detect legitimate but separate components of risky decision-making. Our data do not suggest that the self-report of risk-taking, despite its reported correlation with self-reported smoking, alcohol use, and addiction in the UK Biobank study, captures the dimensions of risky decision-making assessed with the BART although power limitations may have precluded our finding such a relationship.

Notably, the cannabis use sample was the largest of the publicly available samples examined, many times larger than the ADHD and bipolar samples. Since the public samples are highly heterogeneous, it is impossible to make comparative conclusions across disorders. Importantly, the lack of PRS replication does not necessarily imply an absence of shared genetic risk, but rather an inability to detect it with the current samples. It may be relevant that a meta-analysis confirmed that euthymic patients with bipolar I disorder make more risky choices than healthy controls on the Iowa Gambling Task, with an effect size that was small to medium by Cohen’s standard [79]. Moreover, lack of a difference in performance between patients with ADHD and healthy controls on the Cambridge Gambling Task despite differences in real-life risk taking suggests some insensitivity of laboratory tasks to propensity for risk-taking [80]. The positive correlation between BART performance and the categorical diagnosis of prior cannabis use, however, is consistent with the hypothesis that risky decision-making and initiation of substance use share a common genetic link.

This study represents the first genome-wide assessment of heritability of risky decision-making based on BART performance. It benefits from comparing similar objective and complex behavioral measurements in moderately sized, relatively genetically homogenous samples. Suboptimal power is the main limitation of this study. Given the highly complex phenotypes being evaluated, it is likely that a much larger sample is needed to definitively identify the variants and genes associated with risky decision-making and demonstrate an association with these and other psychiatric diagnoses. Further limitations relate to genetic ancestry. The sample is unpowered for meaningful examination of ancestry or sex, and there was an uneven representation of Hispanic ethnicity between the CNP and GOI datasets, which may be responsible for the weaker correlation observed when the discovery set was less homogenous. While principal component analysis shows substantial overlap between the American Hispanic and Caucasian CNP sample, the CNP and GOI datasets are imperfectly matched.

Future analyses would benefit from a more comprehensive approach to ancestry. Regarding the link to psychiatric illness, analyses utilizing public data rely on highly heterogeneous datasets with limited available data. As additional diagnoses and sample numbers expand within the public domain, additional analyses will be possible. Additionally, if available in the future, a replication sample providing independent BART data to confirm the MEGA-PRS would be useful to further validate the findings. Participants were not paid for their performance on the BART in the current study; this could be considered in future studies to enhance motivation. The ability of large population studies to comprehensively capture complex psychological constructs would be facilitated by further understanding of the limitations of self-report data versus objective measures and the development of strategies to align these approaches. Finally, targets for the development of novel psychiatric treatments may be revealed by clarifying the biological bases of common endophenotypes, such as risky decision-making, that may be more therapeutically tractable than categorical disease.

In conclusion, here we demonstrate for the first time that a polygenic score derived from a GWAS of a risk-taking phenotype successfully replicates in a distinct independent sample. Combining the samples, we found that a substantial portion of the variance in performance on the BART was captured by common genetic variation, consistent with the idea that risk-taking behavior is a heritable, highly polygenic trait. A MEGA-analysis GWAS, while not comprehensive due to limited power, produced one significant and one suggestive association in two functionally relevant genes. Finally, shared genetic architecture between BART performance in our sample and categorical cannabis use in a public dataset supports the current model of risky decision-making as a dimensional, intermediate phenotype of substance use disorder.
